# Nutritional and parasitic coprology status, along with the share of school food, to meet daily food consumption among schoolchildren benefiting from school canteens in the community of *Zè*, southern Benin

**DOI:** 10.11604/pamj.2026.54.25.41340

**Published:** 2026-06-03

**Authors:** David Géraud Adomahou, Fernande Honfo, Elvire Assogba, Jaurès Lokonon, Hippolyte Tchango, Inès Mlantahou, Paulin Azokpota, Amoussa Hounkpatin Waliou

**Affiliations:** 1 Institut de Sécurité Alimentaire, Université d'Abomey-Calavi, 03 B.P. 2819, Jericho, Cotonou, Benin,; 2 Laboratoire de Nutrition NUTRI FOODS Lab, Faculté des Sciences Agronomiques, Université d´Abomey-Calavi, 03 B.P. 2819, Jericho, Cotonou, Benin,; 3 Laboratoire des Sciences et Technologies des Aliments, Faculté des Sciences Agronomiques, Université d´Abomey- Calavi, 03 B.P. 2819, Jericho, Cotonou, Benin,; 4 Ecole de Nutrition et des Sciences et Technologies Alimentaires, Faculté des Sciences Agronomiques, Université d'Abomey-Calavi, 03 B.P. 2819, Jericho, Cotonou, Benin

**Keywords:** Schoolchildren, nutrition status, parasitic, school meals, food intake, Southern Benin

## Abstract

**Introduction:**

like many developing countries, Benin has opted for School Feeding Programs to reduce hunger among schoolchildren. The present study assessed the nutritional status of children from school canteens in the commune of Zè (Benin)

**Methods:**

nutritional data, as well as socio-economic characteristics of households, were collected on a sample including 361 schoolchildren. In-depth analysis was performed on 55 schoolchildren, from which blood and stool samples were taken, as well as a 24-h dietary recall investigation to determine the prevalence of anemia, parasitic infection, and the contribution of canteen meals to the coverage of daily needs in micronutrients.

**Results:**

thirteen-point-three percent (13.3%) of children were underweighted with 3% in severe form, and 35.7% of children were stunted, with 8.7% in severe form. The prevalence of anemia was 65.45%, with a predominance for the moderate form (69.4%). No cases of parasitic infection have been detected. As for the school canteen meal, it constitutes an important part of the food intake of schoolchildren, where they ensure at least one-third (1/3) of their daily consumption and 100% of their nutritional iron needs. Among the socioeconomic statuses, only household poverty was significantly associated with the occurrence of anemia.

**Conclusion:**

with a high rate of anemia, an improvement in schoolchildren's diet is recommended. In addition, other in-depth studies are needed to assess the bioavailability of iron and to provide voices to improve the menus served in school canteens in Benin.

## Introduction

Malnutrition is an illness that affects a large majority of the world's population. It is rooted in the inadequacy of macronutrient and micronutrient intakes in relation to the needs of the human body [1]. Indeed, a large proportion of the world's population, 2.82 billion, lacks access to adequate nutrition [2]. The diets of most of these populations consist of plant-based products and are mostly based on a starchy food (cereal, root, or tuber) [3]. This lack of dietary diversity can lead to micronutrient deficiencies that can be detrimental to the health of vulnerable groups of people, especially young school-aged children.

Studies conducted among school-aged children have shown that malnutrition in this group is a public health problem [4]. In 2018, a study of 57 low and middle-income countries by Caleyachetty *et al*. [5] showed that the prevalence of stunting was 10.2% and thinness was 5.5% among school-aged children. The consequences of malnutrition are assessed in terms of the frequency of infectious diseases and mortality among children [6]. Iron deficiency and the resulting anemia are very common and are the number one nutritional deficiency worldwide [6]. In several sub-Saharan African countries, the rate of anemia among school-aged children is between 30 and 50% [7]. In Africa, high prevalences of malnutrition among school-aged children have been reported, reaching 38.7% in southwestern Nigeria [8] and 48.1% in Ethiopia, compared with 54% in South Africa [9].

A study on malnutrition among school-aged children in a Beninese city found a prevalence of underweight at 24% and obesity at 16% [10]. Much of the existing nutritional information is related to preschool children, but few studies have been conducted on school-age children. The health and nutrition of school-age children receive less attention than that of younger children or adolescents. Yet, this stage of life is critical for learning and intellectual development and requires attention [11].

Children as young as three years of age enter a phase of active growth that extends into adolescence [12]. During this dynamic period of physical growth and mental development, poor nutrition results in, among other things, a deficient immune system, increased morbidity, and impaired cognition [11]. All this compromises performance in school and can lead to absenteeism, repetition, or dropout. Each year, such consequences equate to as many as 200 to 500 million school days lost to illness [13]. The undernourished child, the anemic child, or the child who goes to school without having eaten since the previous day cannot have optimal learning in the classroom [14].

The presence of nutritional diseases in children that can affect their physical and intellectual development requires attention to their diet both inside and outside the household. School feeding programs are therefore of significant importance for a large proportion of these children. Aside from the fact that school feeding is a bait for children's attendance and retention in school, it also helps to improve the health and nutritional status of school-aged children [15]. By providing healthy and balanced meals, school feeding programs can improve overall micronutritional status and reduce the prevalence of anemia among elementary schoolchildren and adolescent girls [16]. Benin has not remained behind this dynamic and has been implementing school feeding programs for nearly two decades. Since 2017, Benin, in collaboration with the World Food Program, has been implementing the National Integrated School Feeding Program (PNASI), which reaches nearly 5,500 schools with more than one million schoolchildren [17]. School feeding is thus a field of exit in the fight against malnutrition. Since children consume a large proportion of their daily energy intake at school, measures targeting school feeding can be beneficial in terms of eating behavior, health, and school performance. Therefore, it is necessary to examine the nutritional status of school-age children who are served in school canteens. To contribute to a better understanding of the impact of school canteens on the nutritional situation of schoolchildren, this study aimed to evaluate the nutritional status and parasitic coprology of children beneficiaries of the school feeding program and the comparison between days when children ate school meals and days when they did not, in the Atlantic department, and more specifically in the commune of *Zè*.

## Methods

**Area of study:** the commune of *Zè* is in Southern Benin (Atlantic Department), between 6°32' and 6°87' north latitude and between 2°13' and 2°26' east longitude. It covers an area of 653 km^2^ with a population of 106,913 inhabitants, including 51,381 children aged 6 to 11, of whom 70% are in school [18]. This commune has a total of 95 elementary schools, among which 45 beneficiary of the school canteens program [19]. According to the results of the Global Vulnerability Analysis study on food insecurity conducted in 2017, *Zè* commune is among those in Southern Benin with a poor food consumption rate of over 5% or 7% [20]. According to the same study, consumption of iron-rich foods is rare; only one-third of households had a daily dietary intake of iron. More than half of the households with poor consumption and one-third with borderline consumption do not eat any iron-rich food.

**Type and population of the study:** the study targeted a population of school-aged children between the ages of 5 and 15, enrolled in public primary school in the town of *Zè* and whose parents consented to the study. School of the village of Houezè (district of Tangbo) was chosen because of its proximity to the city, which facilitates rapid transport of blood and stool samples to the laboratory. As this study is part of the evaluation of the bioavailability of iron in school meals, two meals rich in iron and frequently consumed by children were chosen for the occasion: Atassi (a mixture of rice and cowpea accompanied by fried tomato) and Djongoli (cowpea seasoned with oil and mixed with roasted corn flour). Preliminary studies conducted on the nutritional composition of school meals eaten by children support this choice.

### Sampling

**Sample size:** the present study included in the overall framework of the evaluation of iron bioavailability in two meals served in school canteens using stable isotopic markers of iron. The minimum sample size calculated was 361 by using the Schwartz formula with a 5% margin of error, 5% precision, and a prevalence of stunting p = 27% from a study conducted in elementary school in southern Benin (study conducted in 2018 by the NGO We World). A margin of 5% was added to the resulting size. With a standard deviation (SD) of 0.25 (log-transformed difference in fractional iron absorption from a similar previous study of iron absorption in Beninese women), a type I error rate of 5%, and 80% power, a difference in absorption (D) of 25% can be detected with a sample size of 42 children. For a dropout rate of 30%, 55 children were included in the study.

### Selection of schools and participants

**Selection of schools:** the schools were grouped into three categories, considering the level of financial participation of the students and the physical presentation of the kitchens. Thus, a first phase of survey allowed to divide into three categories (low, medium, and high level) all the 45 schools benefiting from the canteens in the commune of *Zè*: The targeted schoolchildren were those aged 5 to 15 years in classes from the 1^st^ year Elementary Course (CE1) to the 2^nd^ year Middle Course (CM2).

***Weighting of enrollments by school and by class:*** the number of children identified by class from CE1 to CM2 and by gender within the 10 selected schools made it possible to determine the number of children to be surveyed for each school, which was obtained by the sampling technique proportional to the size.

***Selection of schoolchildren and households:*** once the number of children (per school, per class, and per gender) was known, children were selected through systematic random sampling using the attendance lists of the students on the day of the survey, taken from the roll call register of each class kept by each teacher before the beginning of the educational activities. The number of schoolchildren by school, class, and gender is consolidated in Table 1.

**Table 1:** details on the number of schoolchildren by school, class, and gender

School's name	Total	Proportion of children selected per school/sample size	Total number to be selected in grade 3	Number of boys to be selected in grade 3	Number of girls to be selected in grade 3	Total number to be selected in grade 4	Number of boys to be selected in grade 4	Number of girls to be selected in grade 4	Total number to be selected in grade 5	Number of boys to be selected in grade 5	Number of girls to be selected in grade 5
Agbodjedo	172	47	15	6	8	19	10	9	13	5	8
Yokpo	163	45	21	10	10	15	8	7	10	6	4
Houeze	248	68	24	14	9	27	15	13	17	8	8
Agbohounsou	96	26	9	5	4	10	7	3	8	5	2
Goulo-Sodji	80	22	8	5	3	9	5	4	5	3	1
Adjan	129	35	11	7	4	16	7	9	8	5	3
Missebo	50	14	5	2	3	5	2	3	3	2	2
Kpatchamey	120	33	13	7	6	13	5	8	7	5	2
Adjrako	98	27	11	6	5	11	6	5	5	3	2
Akouedjromede	159	44	19	10	9	15	9	7	10	5	5
**Total**	1315	360	134	73	61	141	73	68	85	48	38

**Data collection and analysis:** a standardized questionnaire survey of the socioeconomic data of the children's households was conducted. Socioeconomic status was defined by the following parameters or variables: parents´ occupation, household size and number of rooms, type of toilet and water source, main source of water, household appliances available in the house, means of lighting, means of transportation, source of cooking energy used (gas, electricity, wood, charcoal, or other), and parents' occupation. The non-monetary approach is adopted to assess the socio-economic status of the households from which the schoolchildren in the study come. A Composite Poverty Indicator (CPI) was constructed to better understand the situation of each household. The construction of the CPI is based on Multiple Correspondence Analysis (MCA), which is a multidimensional analysis technique for reducing data while retaining the essential information sought. Thanks to the MCA, the weighting coefficients W_m_ were calculated. These are the standardized scores of the categorical indicators on the first factorial axis that describes well-being. Age, sex, anthropometric measurements (weight and height), blood and stool samples, as well as food consumption of the children were collected. The child's age was calculated from the date of birth given by the parents and confirmed with the birth certificate of each child. Height and weight were taken using the WHO standard method [21]. Anthropometric indices (height-for-age, weight-for-age, and body mass index: BMI) were estimated by Z-score calculated with the WHO AnthroPlus software. The classification of children according to the indices is as follows:

**Weight/age (underweight):** this index allowed us to determine the percentage of children with underweight (-3ET≤ Weight/Age <2ET) and severely underweight (weight/age <-3ET). i) Height/age (stunting): this index allowed us to determine the percentage of children with stunting (-3ET≤ height/Age <2ET) and severe stunting height/age <-3ET). ii) BMI/age: this index allowed us to determine the percentage of children with BMI/age (-3ET≤ BMI/Age <2ET) and severe BMI/age (BMI/age < -3ET). - Biological data (thick drop bottles and parasite density): to make the biological diagnosis of anemia, a blood sample was taken from each schoolchild in ETDA tubes. The blood collected slowly turned over to mix the blood and the anticoagulant in the tube. The EDTA tubes were then packed in a cooler equipped with ice for storage, where they were then transported to the Dimed Laboratory in Cotonou town near *Zè*, by car for hemoglobin analysis. The blood analysis provided the hemoglobin (Hb) level, mean corpuscular volume (MCV), and mean corpuscular hemoglobin concentration (MCHC) of each child. Criteria defined by the World Health Organization (WHO) were used to estimate the different prevalence of the main hematological parameters. i) A child was considered to have anemia when his or her Hb <11.5g/dl for children aged 7 to 11 years and Hb<12 g/dl for children aged 12 to 14 years. ii) Mean Globular Volume: a child was considered to have microcytic anemia when his or her MGV<80 fl, macrocytic anemia when his or her MGV>100 fl, and normocytic anemia when 80≤GV≤100fl. iii) Mean corpuscular hemoglobin concentration: a child was considered to have hypochromic anemia when his MCHC<32%, hyperchromic when his MCHC>36%, and normochromic when 32≤MCHC ≤36%. iv) Anemia is considered mild for a hemoglobin level between 10.6 and 11.4 g/dL, moderate for a hemoglobin level between 6.5 and 10.5 g/dL. Hemoglobin level was classified according to the severity of the anemia as shown in Table 2. A stool collection was conducted to determine the parasitic coprology of the children. Stool samples were collected from each child in AKOP tubes, and the tubes were given to the children the day before the collection. Once received from the children, the stools are conditioned with formalin (10 ml). The stool samples were examined directly between slides and coverslips, followed by an examination after parasite concentration by the Willis method. The examination after concentration is preceded by filtration and mixing with another solution. The aim is to determine the parasitic infection in terms of the search for AKOP (A= amoebae, K= amoeba cysts, O= parasite eggs, and P= parasites).

**Table 2:** threshold values for the assessment of anemia (OMS, 2011)

	Anemia based on hemoglobin level in g/l		
**Age category**	**Lower**	**Moderate**	**Severe**
Children 5-11 years	(110-114)	(80-109)	(Below 80)
Children 12-14 years	(110-119)	(80-09)	(Below 80)
**Significance of the anemia situation in terms of public health**			
	**Lower**	**Moderate**	**Severe**
Prevalence of anemia	5.0-19.9%	20.0-9.9%	40% or more

**Food consumption data:** the contribution of canteen meals in covering the daily needs and in the daily food consumption of the children was collected using the 24-h dietary recall method [22]. It consists of a detailed description of all food and beverages consumed, including preparation and cooking methods. Supplement intake is noted, as well as the quantities consumed. For this purpose, a bowl kit with size templates was used to quantify the meals consumed by the child. To estimate the average weight of meals consumed in the canteen, each portion served to the children was systematically weighed, as were the leftovers after consumption. The difference between the weight of the portion served and that of the leftovers made it possible to determine the amount eaten by each child. An average of the amounts eaten was then calculated for the entire sample. This approach made it possible to assess the contribution of canteen meals to meeting the daily nutritional needs of schoolchildren. Then, based on a recent study [23] providing information on the mineral composition of Djongoli and Atassi in the same study area, the average zinc, iron, and calcium intakes provided by the average meals were calculated. These mineral compositions were obtained after analysis of meal samples in the Zurich laboratory in the frame of this study on iron bioavailability in foods consumed by schoolchildren and are recorded in Table 3. The average mineral intakes and recommended intakes for the average age of schoolchildren were used to determine the percentage of micronutrient coverage of the meals consumed.

**Table 3:** mineral composition of Djongoli and Atassi recipes from laboratory analysis

Samples	Iron (mg)	Zinc (mg)	Calcium (mg)	Dry matter (%)	Ash (%)
*Atassi*	1.6^b^±0.6	0.9^b^±0.3	7.0^b^ ±1.5	28.8^b^ ± 1.5	3.1^b^ ±0.5
*Djongoli*	4.6^a^±2.5	1.9^a^±0.6	9.2^b^±0.9	39.4^a^± 0.6	3.9^b^±0.3

Values containing different letters (^a^ or ^b^) for a meal in the same column are significantly different (p < 0.05)

**Statistical analysis:** to address the primary research questions on the relationship between socio-economic status and the nutritional status of children, data were analyzed using R (v4.1.3), with descriptive statistics (means, standard deviations, and proportions) reported. Associations between socioeconomic status, nutritional status, biological parameters, and parasitic infections were examined using chi-square tests for categorical variables and ANOVA or t-tests for continuous variables. The significance level was considered statistically significant. Anthropometric Z-scores were calculated using WHO AnthroPlus. Children were categorized into nutritional status groups following WHO cut-off points: underweight, stunting, and wasting, with severity defined by thresholds. Prevalence rates for each category were estimated. For the socioeconomic status (SES) of households, a Composite Poverty Indicator (CPI) was constructed through Multiple Correspondence Analysis (MCA), which allowed reduction of multidimensional categorical data while retaining essential information on household well-being. Weighting coefficients were derived from standardized scores on the first factorial axis, representing the primary dimension of well-being. The CPI was then used to classify households into two categories according to their socioeconomic status.

**Ethical approval:** this study has received the statistical VISA from the National Statistics Council (N°13/2021/MPD/INSAE/DCSFR) and the favorable ethical opinion of the National Ethics and Health Research Committee of Benin (N° 60 /MS/DC/SGM/CNERS/SA) before its implementation.

## Results

### Forms of malnutrition according to the age groups

***Underweight:*** results revealed that among the 135 children aged 5 to 9 years surveyed, 13.3% were underweight with 3.0% of severe cases for an average weight-for-age z-score of -0.8 ± 0.9.

***Stunting:*** Table 4 presents the stunting according to the age groups of the children. Overall, 35.7% were stunted, with 8.3% of them being severe. The average T/A index is -1.52 ± 1.15 z-scores. The prevalence of stunting by age group was as follows: 22.2% of children aged 5-9 years and 43.6% of children aged 10-14 years. The average BMI of children aged 5 to 9 years was -1.08 and for children aged 10 to 14 years was -1.33 (Table 4). No children in the sample were over weighted.

**Table 4:** prevalence of stunting and average BMI by age group of children surveyed

Age groups (years)	Growth retardation (height/age); n=361 children			BMI
	Means ± SD	% severe (z-score < -3)	% overall (z-score < -2)	Means ± SD
5 - 9 (n=135)	-1.06 ± 1.17	3.7	22.2	-1.08 ± 0.85
10 - 14 (n=225)	-1.79 ± 1.05	10.7	43.6	-1.33 ± 0.88
15 (n=1)	-3.48 ± 0	100	100	-
Total (n=361)	-1.52 ± 1.15	8.3	35.7	-1.24 ± 0.87

SD: standard deviation; BMI: body mass index

**Prevalence of anemia:** the prevalence of anemia was 65.5% among schoolchildren, with 47.2% for girls and 52.8% for boys. Anemia was more prevalent among children aged 7 to 11 years (29.0%) compared to children aged 12 to 15 years (7%) (Table 5). There was no significant difference in the prevalence of anemia by age and sex. As shown in Figure 1, moderate anemia is the most prevalent form among schoolchildren (69.4%). Figure 2 shows the forms of anemia according to their erythrocyte count. Normochromic microcytic anemia was the most common form of anemia among schoolchildren, with 42.0%. Normocytic normochromic anemia was 28.0%. Hypochromic microcytic anemia was identified as 22.0% and hypochromic monocytic as 8.0%.

**Table 5:** prevalence of anemia among schoolchildren

Variables	Anemia; n (%)	No. anemia; n (%)	Total; n (%)	P-value
**Sex**				**0.717**
**Female**	17 (47.2%)	8 (42.1%)	25 (45.5%)	
**Male**	19 (52.8%)	11 (57.9%)	30 (54.5%)	
**Year**				**0.738**
7-11	29 (80.6%)	16 (84.2%)	45 (81.8%)	
12-15	7(19.4%)	3 (15.8%)	10 (18.2%)	

**Figure 1:**
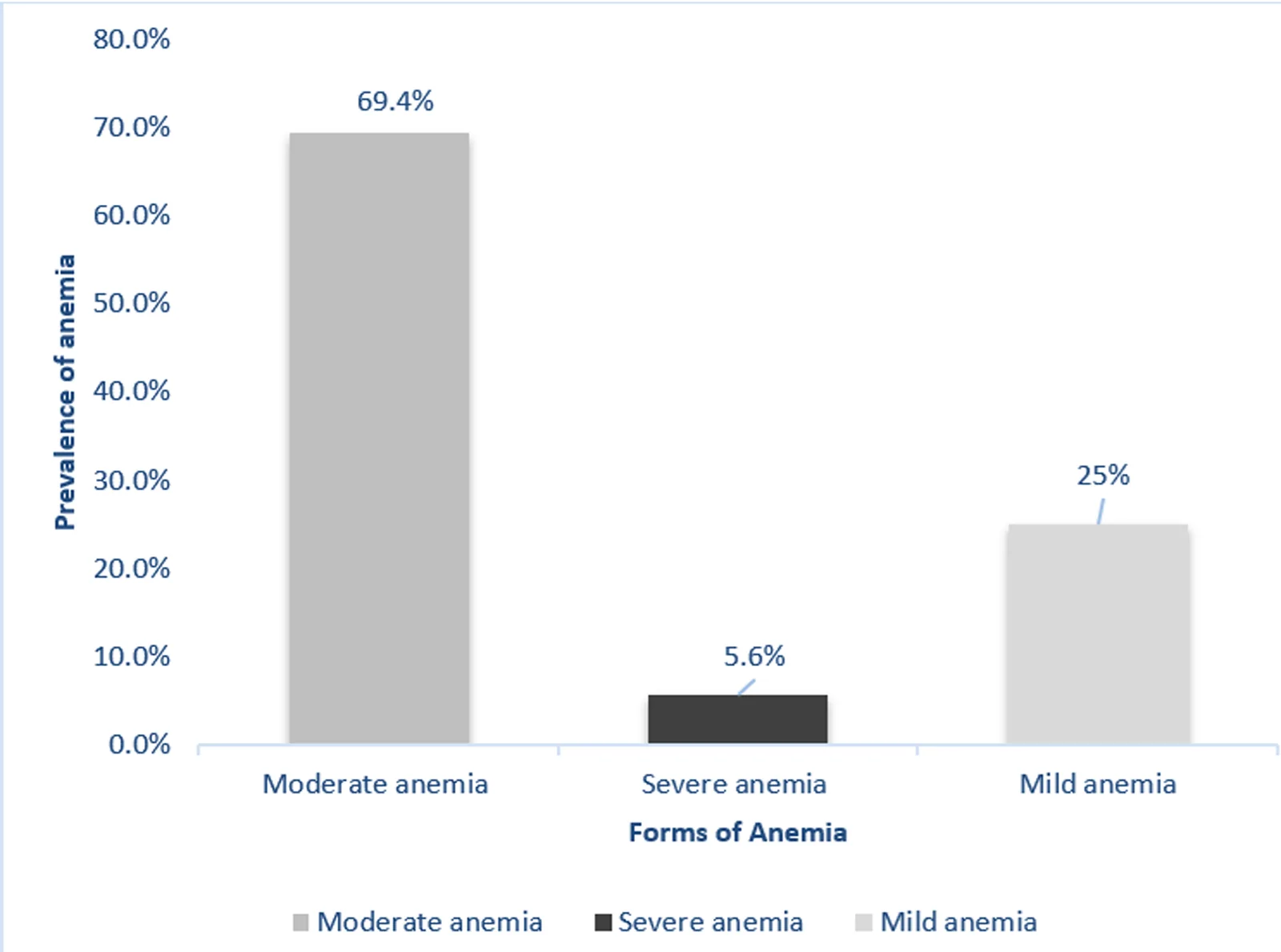
forms of anemia among schoolchildren who benefit from the school canteens

**Figure 2:**
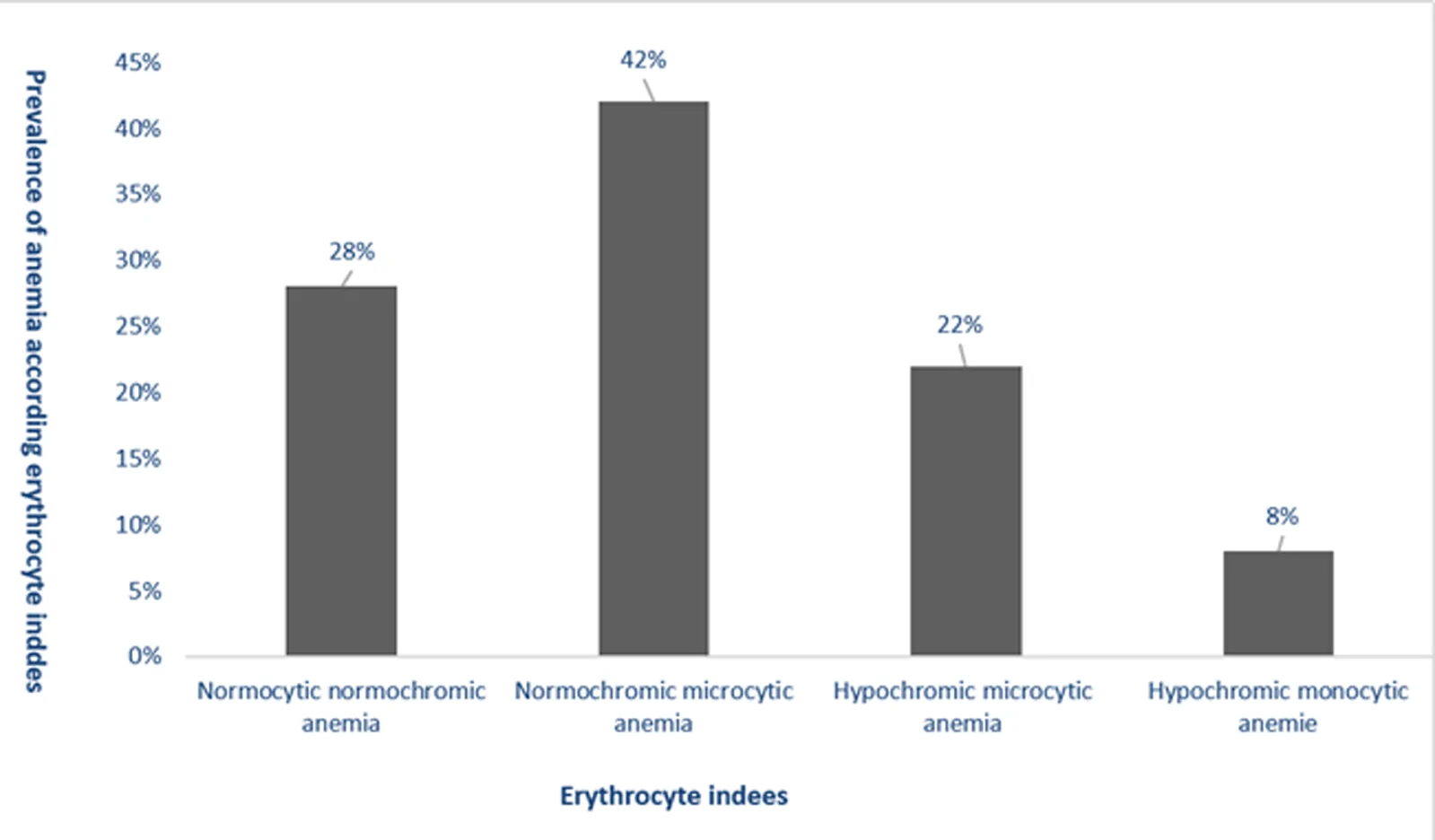
prevalence of anemia according to erythrocyte indices

**Socio-economic status:** results showed the distribution of schoolchildren's households according to the socio-economic status of their households. Two classes have been determined: the poor and the wealthy. Most children surveyed (n= 55), 81.8%, come from poor households. The non-poor represent a minority of the population surveyed, 18.2%.

**Socio-economic and nutritional status relationship:** within the different nutritional status screening in this study, only a significant relationship between anemia and household poverty was observed (p-value= 0.017) (Table 6). Thus, schoolchildren from poor households were more susceptible to suffering anemia than those from wealthy households.

**Table 6:** relationship between anemia and socioeconomic status

Variables	Anemia		
Predictors	Odds ratio	95% CI	P-value
Socioeconomic status (poor)	0.12	0.02 - 0.61	0.017*

* Significant at p < 0.05; CI: confidence interval

**Parasitic coprology of schoolchildren:** after analysis of stools, no child showed a parasitic infection. Only the presence of yeast was noted.

**Share of school food in the daily food consumption of children:** Figure 3 shows the effective share of school feeding in children's daily consumption; 38.2% of the canteen meals consumed by children represent one third of their daily consumption. However, it was also found that for 45.6% of children canteen meals could exceed one third, or even slightly more than half of their daily consumption.

**Figure 3:**
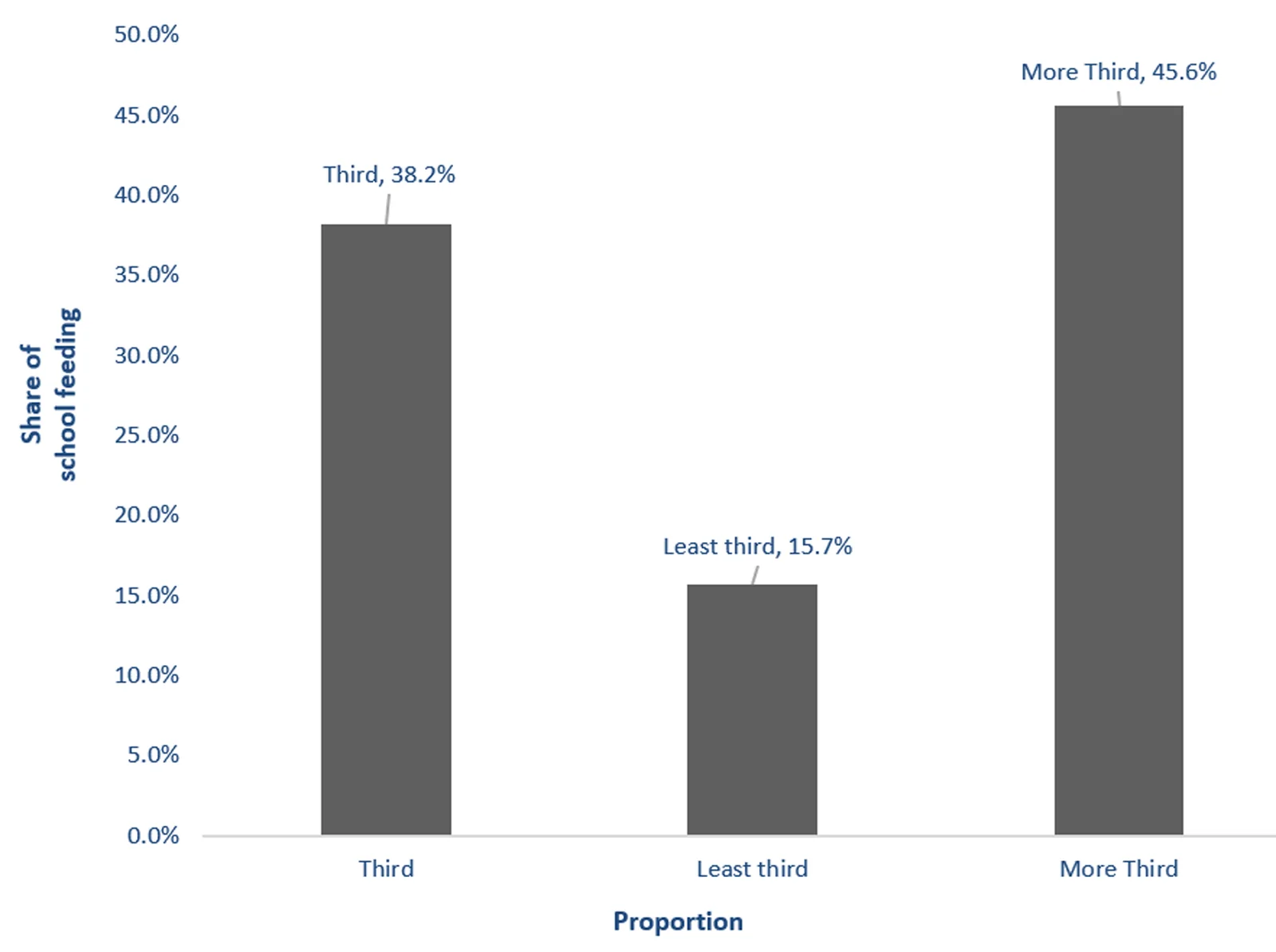
share of school feeding in children's daily intake

**Comparison between food intake on canteen days and ordinary days:** a significant difference was observed between the intakes on ordinary days (ingested at home) and those on canteen days (p-value= 4.389e-11) (Table 7). Average intake on canteen days was more than 325 g higher than the average intake on regular days.

**Table 7:** comparison of food intake on canteen days and ordinary days among the schoolchildren surveyed

	Estimate	Confidence interval	P-value
**Incorporated school days and non-working days**	325.73	233.54 - 17.93	4.389e-11***

*** = significant degree

## Discussion

Several types of malnutrition were observed in the school-age population, namely underweight, stunting, and BMI for age. Stunting is the most noted form of malnutrition, with a percentage of 37.5%. This result was higher than that found by Achouri *et al*. in the Kenitra region of Morocco, where the prevalence was 10.9% [24]. This difference could be explained by contextual disparities between the two populations studied: in particular, socioeconomic conditions, household food security, diet quality and diversity, and access to health and sanitation services. Prevalence of underweight was 13.3%. This result was comparable to that obtained by El Hioui *et al*. in 2008 in the city of Kenitra, where the prevalence of underweight was 12.7% [25]. In contrast, our finding was higher than those observed in Constantine (Algeria) by Oulamara *et al*. in 2006, where the prevalence was 1.6% in children aged 8 to 12 years [26]. However, the opposite result was observed in Congo Brazzaville by Mbemba *et al*. who´s underweight exceed 45.0% in children aged 6 to 14 years [27] and by Makoutode *et al*. in 2022 in Benin, where a nutritional study among schoolchildren in the urban area of Ouidah city was 36.9% for underweight, almost three times as much; and the overweight for the same study was 0.6% [28]. The difference observed in overweight could be explained by the type of environment: urban for the Ntandou *et al*. study and rural for our study; the food habit (rich in fat and superfluous foods for the urban and peri-urban environment) and the lifestyle (sedentary in urban areas and physical and active in rural areas) [29].

The prevalence of anemia among schoolchildren was 65.5%, higher than the threshold of the WHO classification, and appears severe. This rate was higher than that obtained by Savadogo *et al*. in 2014 in Burkina-Faso and Kokoré *et al*. in 2013 in Côte d'Ivoire, with respective prevalence of anemia of 35.2% and 30.8% in school-age children [30,31]. The present study reveals that boys (52.8%) were more vulnerable to anemia compared to girls (47.2%). However, no statistically significant association was found between anemia prevalence and the sex or age of the children. The male predominance in our sample may partly explain this observation. This observation is in line with that of El Hioui *et al*. in 2009, according to a study carried out on children of the same age group in Morocco [32]. Among the forms of anemia identified, moderate anemia was the most observed form in anemic schoolchildren, and this result was similar to those obtained by Kokoré *et al*. in 2013 in Morocco and Sudhagandhi and colleagues in 2011 in India on children of the same age group [31,33]. According to VGM erythrocyte constancy, microcytic anemia was most prevalent in children. This could be explained by iron deficiency among schoolchildren due either to insufficient dietary iron intake, digestive malabsorption, or excessive iron loss [34]. Among others, normochromic microcytic anemia was the most common form of anemia in children. This was in contradiction with the results found by Kokoré and colleagues, where the most present form in children was the normocytic hypochromic form [31]. In a study conducted in Morocco, a positive correlation was shown between serum ferritin and hemoglobin concentrations, which explains that a significant proportion of anemia may be related to iron deficiency. In this study, no association of parasitosis can be made with anemia since no child here has a parasitic infection.

Most children surveyed were from poor households (81.8%). Socioeconomic status plays an important role in the occurrence of anemia in the schoolchildren surveyed. Indeed, children from poor households were 8 times more exposed to the occurrence of anemia. This result confirmed those found by El Hioui *et al*. in 2009 and Ndour *et al*. in 2019 among school-age and preschool children [32,35]. According to their results, the prevalence of anemia decreases with improving economic status. Regarding the proportion of school canteen meals, most of the schoolchildren surveyed consume at least one-third (1/3) of their daily rations at school. This meets the requirement of the WFP, which, through the food basket, provides one-third of the daily needs of children. This normal rate of (1/3) in some cases was equivalent to half the daily food intake of schoolchildren [15]. This situation reflects the vulnerability of households and their poor access to food.

**Limitations:** the limitation of this study lies in the lack of assessment of schoolchildren's malaria status since other studies conducted on school-age children revealed a significant association with malaria. Determining iron deficiency could improve the diagnosis of anemia. Furthermore, the small size of our sample does not give a clear picture of the overall anemia situation in school-age children.

## Conclusion

The present study carried out in the commune of *Zè* provides information on the nutritional status of school canteens children. From a nutritional standpoint, stunted growth was the most common form of malnutrition among children, and more than half of the schoolchildren surveyed were anemic. Anemia was significantly associated with socioeconomic status but was independent of gender and age. Parasitological examination of stool samples revealed no intestinal parasitic infections; only the presence of yeast was observed. School canteen provided an important part of daily feeding, such as two-thirds of the daily food intake of the children. Policies targeting school nutrition should be promoted to control nutritional diseases that can affect the well-being of schoolchildren.

***What is known about this topic***

- *The daily ration provided to schoolchildren is specifically designed to provide schoolchildren with several micronutrients that are essential for their health and growth and to reduce deficiencies that could affect their schooling; the daily ration served in school canteens provides 42% of the daily caloric needs, and notably: 100% of the daily iodine needs, 48% of the daily iron needs, and 19% of the daily vitamin A needs for a schoolchild*.

***What this study adds***

- *This study provides one of the first pieces of evidence and data on the nutritional and coprological status of schoolchildren receiving school meals since the advent of the National Integrated School Feeding Program (NISFP) in Benin in 2017;*
- *It also shows the prevalence of anemia among schoolchildren since the advent of NISFP, and the relationship between anemia and the socioeconomic status of schoolchildren's households;*
- *It also shows the relationship between anemia and the nutritional status of children*.
